# Rescue of kidney function in a toddler with anti-GBM nephritis

**DOI:** 10.1093/ckj/sfs146

**Published:** 2012-12

**Authors:** Anna Bjerre, Kolbjørn Høgåsen, Jon Grøtta, Helge Scott, Trine Tangeraas, Christina Dörje

**Affiliations:** 1 Department of Pediatrics, Division for Specialised Medicine, Oslo University Hospital, Rikshospitalet, Oslo, Norway; 2 Department of Immunology and Transfusion Medicine, Sykehuset Innlandet Trust, Lillehammer, Norway; 3 Department of Pediatrics, Sykehuset Innlandet Trust, Elverum, Norway; 4 Department of Pathology, Division for Diagnostics and Intervention, Oslo University Hospital, Rikshospitalet, Oslo, Norway; 5 Department of Transplant Medicine, Oslo University Hospital, Rikshospitalet, Oslo, Norway

**Keywords:** anti-GBM nephritis, kidney function, RPGN, toddler

## Abstract

Anti-glomerular basement membrane (anti-GBM) nephritis is rare in childhood with few published cases. We report a 19-month-old boy with rapidly progressive glomerulonephritis (RPGN) due to anti-GBM nephritis. Treatment was started under 2 weeks after presentation and included plasma exchange, intravenous high-dose methylprednisolone, intravenous cyclophosphamide and mycophenolate as mainstay medication. The treatment was rapidly effective with immediate decrease in anti-GBM titres and plasma creatinine. Three years after presentation, the boy has normal kidney function, blood pressure and no residual disease. The successful outcome was likely due to the rapid recognition of the anti-GBM antibodies as the cause of RPGN and aggressive primary treatment.

## Background

Anti-glomerular basement membrane (anti-GBM) disease is characterized by rapidly progressive glomerulonephritis (RPGN) caused by autoantibodies against the α3-chain of type IV collagen in the GBM [1]. The disease is extremely uncommon in childhood; in a report form a tertiary centre, only four cases were identified over 25 years [2]. Since 1983, there have been five reported cases in children <6 years of age, the youngest 11 months old [3–7]. Of these, only one recovered completely, one developed moderate kidney failure, two progressed to end-stage renal failure and one died, reflecting the diverse prognoses of the disease.

In adults, rapid diagnosis and intervention have been reported to correlate with an improved outcome [8, 9]. There is scarce data concerning treatment and outcome in childhood. We report a case of a 19-month-old boy with severe disease with RPGN, early recognition and an excellent outcome.

## Case report

A 19-month-old boy was admitted to a local hospital after 5 days history of vomiting and temperature above 39°C. A urinary tract infection was initially suspected due to the presence of *Escherichia coli* >100 000 colony-forming units (CFU)/mL in a urine specimen. His urine became cola-coloured with red cell cast formation, and plasma creatinine increased rapidly. Evaluation for RPGN was performed. The enzyme linked immuno assay (ELISA) test for anti-GBM (Orgentec, Alegria) was strongly positive with >200 U/mL (ref. <20). The immunofluorescence (IF) test for anti-GBM on primate kidney tissue slides (Inova) was positive with a titre of >40 (ref. <10). Antinuclear antibodies (ANA) ELISA (‘8 screen’, Phadia), antineutrophil cytoplasmic autoantibodies (ANCA) IF (Inova), myeloperoxidase (MPO)-ANCA ELISA and proteinase 3 (PR3)-ANCA ELISA (both Orgentec, Alegria) tests were all negative. C3 was 1.5 g/L (0.67–1.29) and C4 was 0.29 g/L (0.13–0.32 g/L) (see Table 1 for details).

**Table 1.** Summary of laboratory values^a^

	Day 5	Day 10	3 months	3 years
Haemoglobin (10.5–14 g/dL)	8.7	6.8	12.1	12.8
Leucocytes (6–17 × 10^9^/L)	7.6	8.9	6.6	5.3
Platelets (100–450 × 10^9^ /L)	538	689	506	320
CRP (<1 mg/L)	218	322	<1	<1
Albumin (36–48 g/L)	NA	23	42	45
Creatinine (23–37 µmol/L)	96	150	25	33
Anti-GBM ELISA (U/mL)	>200^b^	167^c^	<10^c^	<10^c^
Anti-GBM IF	>40^b^	>5^c^	>5^c^	<5^c^
ANA^d^	Neg	Neg	NA	NA
ANCA^d^	Neg	Neg	NA	NA
C3 (0.77–1.95 g/L)	1.5	1.37	NA	NA
C4 (0.07–0.40 g/L)	0.29	0.27	NA	NA
Protein/creatinine ratio(<30 mg/mmol)	NA	389	104	17

^a^The IF test for anti-GBM on primate kidney tissue slides (Inova) was positive with a titre of >40 (ref. <10). NA, not analysed.

^b^The ELISA test for anti-GBM (Orgentec, Alegria) was strongly positive with >200 U/mL (ref. <20).

^c^Wieslab, ref. <10, and kidney tissue slides >5 (ref. <5).

^d^ANA ELISA (‘8 screen’, Phadia), ANCA IF (Inova), MPO-ANCA ELISA and PR3-ANCA ELISA (both Orgentec, Alegria) tests.

He was transferred to a tertiary centre for a kidney biopsy. On admission, he was in poor clinical condition with peripheral oedema, blood pressure 116/55 mmHg, body temperature of 39.5°C and deteriorating kidney function (Table 1). Blood sampling showed a haemoglobin of 6.8 g/dL (10–14.0 g/dL); platelets 689 × 10^9^/L (150–450 × 10^9^/L); leucocytes 8.9 × 10^9^/L (6.0–17.0×10^9^/L); albumin 23 g/L (36–48 g/L); plasma creatinine 150 µmol/L (15–31 µmol/L); urea 13.8 mmol/L (3.2–8.0 mmol/L); C-reactive protein (CRP) 322 mg/L (< 5 mg/dL); negative ANA and ANCA and normal C3 and C4. Anti-GBM ELISA was 167 U/mL (Wieslab, ref. <10), anti-GBM IF titre >5 (Wieslab, ref. <5). The spot urine total protein/creatinine ratio was 389 mg/mmol.

**Fig. 1.**
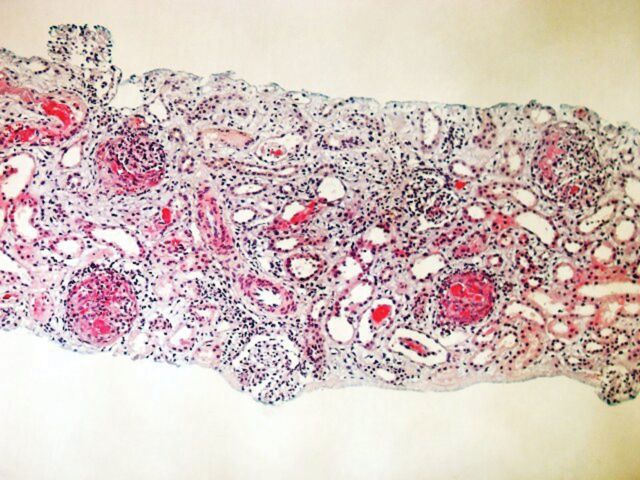
HES (haematoxylin, eosin, saffron)-stained section from kidney showing fibrinoid necrosis in four of seven glomeruli and interstitiel oedema. Original magnification ×120. 225 × 169 mm (72 × 72 DPI).

The percutaneous kidney biopsy contained 17 glomeruli, 13 of them had crescents and/or fibrinoid necrosis with granulocyte infiltration. Tubuli were atrophic with interstitial oedema and bleeding and lymphocyte infiltration was noted (Figure 1). Immunohistochemistry performed on the formalin-fixed material showed a weak global linear staining of GBMs for IgG, but not for C3.

**Fig. 2.**
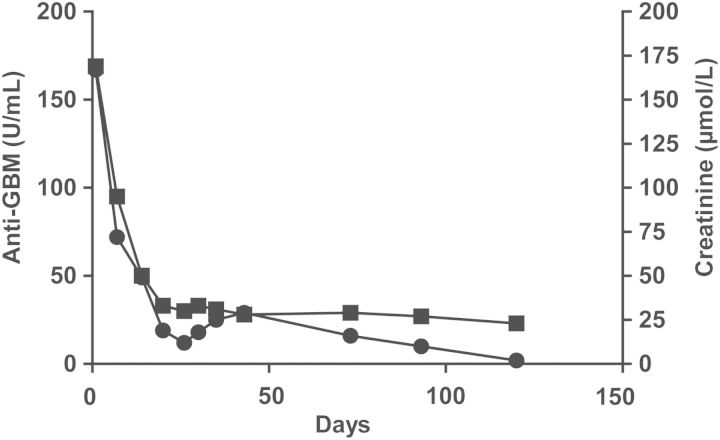
Pattern of plasma creatinine and anti-GBM levels during treatment. Black box, creatinine; black circle, anti-GBM.

Treatment consisted of daily plasma exchange (PE) for 5 days with exchange of 50 mL/kg plasma volume against human plasma, followed by alternate day PE against 4% albumin in Ringer solution, with a total of 13 sessions. As the extracorporal volume exceeded 10% of total blood volume, the equipment was primed with 4% albumin. Methylprednisolone (15 mg/kg) was given for 3 days followed by prednisolone 2 mg/kg/day and thereafter, cyclophosphamide, 10 mg/kg infusions every 4 weeks, with a total of six sessions. He developed hypertensive crisis, which was reversed with medical treatment. Following the initial treatment, he was started on mycophenolate. In addition, he received captopril due to proteinuria and hypertension. Plasma creatinine and anti-GBM titres decreased rapidly (Figure 2). Three months after the first admission, his plasma creatinine was 25 µmol/L and albumin 42 g/L; his plasma creatinine was normal, while low-grade proteinuria was persistent. All medication was gradually tapered and ceased 2 years after admission. Three years later, his condition was excellent, he had regained his growth percentile, his blood pressure was normal (100/55) with normal kidney function, plasma creatinine 33 µmol/L and cystatin C 0.73 mg/dL, and anti-GBM antibody levels remained undetectable (Table 1).

## Discussion

This case demonstrates that rapid diagnosis and prompt aggressive treatment of anti-GBM nephritis can reverse a serious condition with a potentially poor prognosis. This child had a fulminant clinical course with extensive crescenteric glomerulonephritis (>75% of glomeruli affected); treatment started in <14 days after the presenting symptoms due to the early recognition of the disease.

Currently, there is no consensus on how to define the early recognition of RPGN. We believe that reaching a diagnosis within 2 weeks of symptoms appearing would be defined as early recognition. Such a timeframe could provide a basis to compare the outcome of different treatment trials.

Crucial for the diagnosis is the availability of rapid laboratory diagnostics in children with RPGN. As in our case, tests for anti-GBM are not standardized, as reflected in the different reference values from the two laboratories. However, the two ELISA tests and IF tests used in this case showed reliable results. The sensitivity and availability of the present ELISA methods are excellent, so both laboratories were able to perform screening with ELISA and confirm positive results with IF.

Because anti-GBM nephritis progresses rapidly and is rare in childhood, randomized trials are not an option. Treatment recommendations are therefore often extrapolated from studies in adults. Merkel *et al*. [8] published the outcomes of 35 adult patients with anti-GBM nephritis and evaluated prognostic factors. This retrospective survey concluded that the earlier therapy is started, the better the outcome, especially in patients with increased creatinine and crescent formation (>50%). Shah and Hugghins [10] reviewed a total of 84 adult cases and one paediatric case and concluded that prompt treatment improves survival. Most of these patients had been treated with PE and immunosuppressants [10].

The outcome after different treatment regimens was analysed in another retrospective survey in 221 adult patients in China: (i) combination of PE and immunosuppression, (ii) steroids and cytotoxic agents and (iii) steroids only. The conclusion was that PE, in conjunction with steroids and cyclophosphamide, had an overall beneficial effect for both the patient and kidney survival [11].

We performed the first five PE sessions daily to achieve a rapid antibody removal, shown as an abrupt drop in anti-GBM antibody levels. Initially, human plasma was used as exchange fluid to reduce bleeding complications after the procedures of kidney biopsy and dialysis catheter implantation. PE therapy was maintained until kidney function was stabilized and anti-GBM levels remained low, as often seen after 14 days [12]. The anti-GBM antibody levels remained low thereafter, indicating efficient removal. In addition, kidney function improved. Mycophenolate has emerged as an efficient drug in treating vasculitis, but data are lacking concerning anti-GBM nephritis. Takeda *et al*. [13] showed, in a rat model of anti-GBM nephritis, a significant reduction in crescent formation when treated with mycophenolate. In a recent publication, successful eradication of antibodies in a patient with therapy-resistant circulating anti-GBM antibodies was seen after treatment with mycophenolate, but, so far, few data exist in the literature concerning anti-GBM nephritis and mycophenolate [14].

In conclusion, anti-GBM nephritis can also occur in small children, and early diagnosis is imperative to rescue kidney function.

## Conflict of interest statement

None declared.
